# Effect of *Bacillus subtilis* on corrosion behavior of 10MnNiCrCu steel in marine environment

**DOI:** 10.1038/s41598-020-62809-y

**Published:** 2020-04-01

**Authors:** Y. S. Wang, L. Liu, Q. Fu, J. Sun, Z. Y. An, R. Ding, Y. Li, X. D. Zhao

**Affiliations:** 1 School of Ocean, Yantai University, Yantai, 264005 China; 2 School of Civil Engineering, Yantai University, Yantai, 264005 China; 3 Qingdao Branch of Naval Aeronautical Engineering Academy, Qingdao, 266041 China

**Keywords:** Marine chemistry, Corrosion

## Abstract

Bacillus widely exists in wet natural environment such as soil, water and air, and is often studied as one of representative microorganisms for microbiologically influenced corrosion(MIC) research. In this paper, the growth curve of *Bacillus subtilis* isolated from marine environment was determined by turbidimetry and its effect on corrosion behavior of 10MnNiCrCu steel was studied by open circuit potential, AC impedance, polarization curve and scanning electron microscopy(SEM). The results showed that with the change of the growth curve of *Bacillus subtilis*(*BS*), the open circuit potential(*E*_*ocp*_) shifted positively and then negatively, and the charge transfer resistance shown by AC impedance was much lower than that of the sterile system, increasing first and then decreasing. The polarization curves showed that the corrosion current density in BS medium was obviously higher than that in sterile system. The corrosion morphology observation showed that although a biofilm by BS developed on the steel surface, the localized corrosion of 10MnNiCrCu steel was aggravated due to the acidness of the metabolite itself and the biofilm with access for electrolyte ions.

## Introduction

MIC is a major problem involved in many industries such as natural gas, fuel pipeline and water resources^1,2^. All materials in contact with natural environment such as water, soil and moist air are subjected to different degrees of MIC^3^. The essence of MIC is that due to the activity of microorganisms, the electrode reaction in the corrosion process is changed and complex electrochemical process of material corrosion is accelerated or inhibited^4^. Studies have shown that extracellular polymer substances(EPS) produced by bacteria are directly related to the MIC process. EPS usually contains various organic groups, such as fatty acids, proteins and polysaccharides, which can change surface charge, wettability and free energy. In general, EPS changes the steel surface for bacterial adhesion and survival, and develops an overall and inhomogeneous biofilm on the metal surface^5,6^.

*Bacillus sp*, as one of the most widespread microbial species, is ubiquitous in various natural environments^7^. Its metabolites contain cyclic peptides, antibiotics, polyglutamic acid, polyaspartic acid and polypolysaccharide, and can form biofilms with spores^8^. Research showed that EPS produced by Bacillus was the main component of the biofilm formed on the metal surface and provided the biologically active compound which affected the corrosion process^9^. More and more attention has been paid to the corrosion effect of Bacillus on metals, but the mechanism is still unclear, and there is still much controversy. J. A. Rajasekar^10^ proposed that *Bacillus cereus* accelerated microbial corrosion of 2024 aluminum alloy due to the main function of peroxide. The accumulation of corrosion products led to the destruction of the oxide layer on the surface of aluminum alloy, thus forming a less compact biofilm and alumina layer on the surface of aluminum alloy accelerated pitting corrosion. Qing Qu^11^ studied the effect of *B. subtilis* C2 (*BS*) on the corrosion behavior of cold rolled steel in artificial seawater. The results showed that the pH and open circuit potential of cold rolled steel in BS-containing solution decreased significantly. It was confirmed that the reason for the increase of corrosion of cold rolled steel in the presence of *B. subtilis* C2 was the organic acid produced in the metabolic activity of BS in earlier stage, which accelerated the corrosion. Masoumeh Moradi^12^ studied the effect of *Bacillus Vietnam* on the corrosion behavior of different alloys. The existence of protease was confirmed by LC-MS/MS analysis, which indicated that the activity of protease could prevent the corrosion of copper alloys.

It was proposed that protease could coordinate with copper ions according to its molecular structure and bind with water molecules, thus reducing the oxygen utilization rate of cathodic reaction and inhibiting the corrosion process of copper-based alloys. The research of N. Bolton^13^ showed that *Bacillus pumilus* could increase the corrosion rate of galvanized steel but had no effect on the corrosion rate of carbon steel. It was believed that the adhesion of microbial metabolites (including enzymes on metal surface) was the mechanism leading to corrosion. R. F. Jack^14^ suggested that the microbial membrane formed by *B. subtilis* promoted the corrosion of carbon steel, and that the metabolites in the membrane might be the cause of accelerated corrosion.

Because of the difference of strains and materials, the above work mostly describes the corrosion phenomena in the study, and the proposed corrosion mechanism also varies. However, there is a consensus that the biofilm formed on the metal surface has an important impact on the corrosion of microorganisms. Therefore, by comparing the effect of biofilm on metal corrosion on metal surface, we can understand the important role of biofilm in metal corrosion process more clearly.

The 10MnNiCrCu steel is used as a structural material in the shipbuilding industry because of its superior mechanical properties. However, the corrosion of materials under the influence of various microorganisms will inevitably be involved in the marine ships due to their service environment. In this work, *B. subtilis* was isolated and purified from marine environment. The corrosion mechanism of 10MnNiCrCu steel in *Bacillus* and sterile system was discussed in order to provide a new theoretical basis for the study of MIC.

## Experimental

### Materials

The main chemical compositions of 10MnNiCrCu steel in the experiment are C(<0.12%), Si(0.40–0.70%), P(<0.015%), S(<0.010%), Mn(0.70–1.10%), Ni(0.50–1.10%), Cr(0.60–0.90%), Cu(0.40–0.60%), Ti(<0.020%) V(<0.080%) and Fe balance. The 10MnNiCrCu steel sample was processed into two specifications. The samples used for electrochemical measurements were machined to sheet electrodes with an exposed surface of 10 mm × 10 mm, and the rest was sealed with epoxy resin in a cylindrical cavity. A copper wire was soldered to each sample for electrochemical measurements. The samples used for morphology observation were 10 mm ×10 mm × 10 mm cubes. Before the experiment, the working surface of each sample was polished with 240 to 1200# sandpapers, then rinsed with distilled water and degreased with acetone. The samples were placed in a deoxygenation chamber and tested after UV disinfection for 30 minutes.

### Bacteria and culture

The *Bacillus subtilis* used in the experiment was taken from the seawater near Laizhou Bay. The composition of the medium used was as follows: 10 g peptone, 3 g beef extract, 5 g sodium chloride in 1 L deionized water, and the pH value was adjusted with 1 mol·L^−1^ NaOH(7.0 ± 0.2). The medium was autoclaved at 121 °C for 20 min. A single BS solution was prepared by introducing the enriched bacteria into the sterile medium at a volume ratio of 1:10. The inoculated medium was used as the experimental system of bacteria and cultured in a constant temperature incubator (28 °C). In addition, the same amount of sterile liquid medium was used as the sterile system.

The growth curve of *Bacillus subtilis* was determined by turbidimetry. The purified *Bacillus subtilis* was cultured in a constant temperature incubator for 7 days. According to the pre-set time gradient, the absorbance at 600 nm wavelength was measured by TU-1810 ultraviolet-visible spectrophotometer. The value was recorded three times and taken average as the characterization of the bacterial quantity during a certain period of growth.

### Electrochemical measurements

The electrochemical workstation(PARSTAT2273, USA) was used for electrochemical analysis. The 10MnNiCrCu steel is used as working electrode, a platinum sheet (10 × 10 × 0.1 mm) used as the counter electrode, and a saturated calomel electrode (SCE) as the reference electrode. After stabilization of the system, the open circuit potential was measured. Electrochemical impedance spectroscopy (EIS) was carried out in the frequency range of 10^−2^–10^5^ Hz at intervals of 1 day and the amplitude of the sinusoidal voltage signal was 10 mV. The Data were processed using ZSimpWin software to analyze the structure of the equivalent circuit and the parameters of the components. The electrochemical polarization curve was tested and scanned on the 7th day of the experiment. The scanning rate is 0.33 mV/s, and the scanning range is ±350 mV relative to the open-circuit potential. The data are analyzed by C-view software.

The samples for corrosion morphology observation were immersed in the above-mentioned bacterial culture solution and sterile culture system. After 7 days, the specimens were taken out, washed with distilled water, degreased and dried with acetone, sprayed with palladium, and observed under scanning electron microscope(SEM).

## Results

### Growth curve

**Figure 1:**
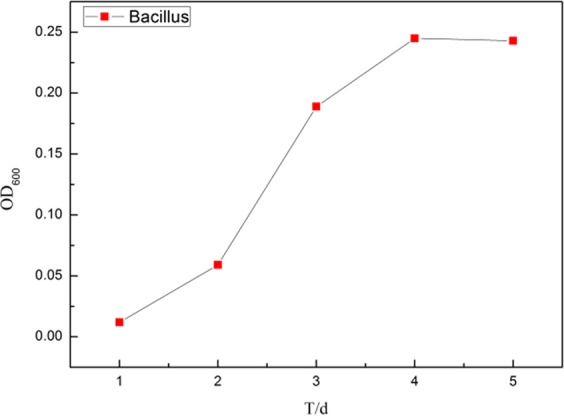
Growth curve of *Bacillus subtilis* with time.

Figure 1 shows the growth curve of BS by turbidimetry. It can be seen that bacteria grew slowly in 1–2 days. The reason was that the bacterial metabolic system needed to adapt a new culture after its inoculation. At the same time, enzymes, coenzymes and other metabolic intermediates were produced^15,16^, thus the bacteria were in a lag phase. During 2–3 days, the bacteria entered the logarithmic growth phase after the preparation of the adjustment period. Sufficient material base was provided for the growth of microorganisms in this period, the external environment was also in the best state, and the bacteria grew fast.

After 4 days, the bacteria entered a stable phase, in which the number of bacteria was the largest. The cell proliferation reached a stable stage due to the consumption of nutrients or growth inhibited by the accumulation of metabolites, and the growth and death rate were balanced. The determination of growth curve of *Bacillus subtilis* was supposed to give theoretical basis for the analysis of subsequent experiments.

### Open circuit potential

**Figure 2:**
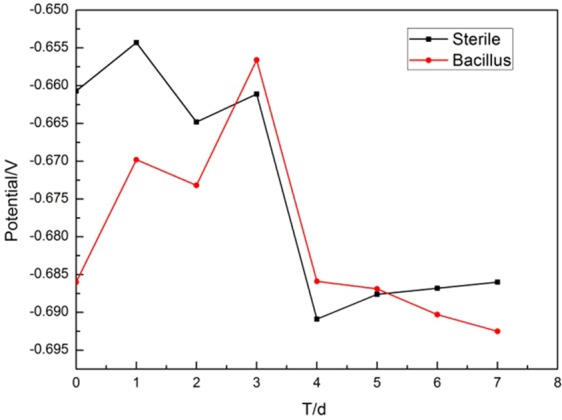
*E*_*ocp*_ of 10MnNiCrCu steel in media with and without BS over time.

It is seen from Fig. 2 that *E*_*ocp*_ in both sterile and Bacillus systems has shifted positively during the first day. It indicated that a product film developed in the early stage of the experiment due to the material of 10MnNiCrCu steel itself, which changed the state of the electrode surface and protected it temporarily^17^. Since then, the *E*_*ocp*_ in sterile system shifted negatively due to oxygen absorption corrosion and stabilized after 5 days^18^. According to the growth curve of bacteria, bacteria proliferated and adsorbed on metal surface in the logarithmic growth phase within 2–3 days.

The corrosion slowed down, the *E*_*ocp*_ moved positively and the proliferation of bacteria consumed dissolved oxygen in the system. The potential shifted negatively within 3–4 days as a result of partial destruction of the formed product film. The bacterial metabolism was vigorous in Bacillus system during this period. The formation of organic acids in metabolites adsorbed on metal surface was conducive to the destruction of corrosion product film and accelerated the corrosion rate of metals. After 4 days, the number of bacteria reached a relatively stable state. The *E*_*ocp*_ of 5d-6d was obviously lower than that of sterile system, which indicated that BS accelerated the corrosion of 10MnNiCrCu steel to a certain extent.

Compared with sterile solutions, the decrease of *E*_*ocp*_ in BS-containing system can be attributed to the formation of biofilm, which inhibits the diffusion of cathode depolarizers (e.g., oxygen, hydrogen ions), leading to a reduction in cathode reduction potential. Therefore, according to the theory of mixed potentials, *E*_*ocp*_ decreases in the presence of BS biofilm. The formation of biofilm in the early stage slows down the corrosion rate. The apparent negative shift of the potential in the later period is the result of a comprehensive effect. With the growth of bacteria and further corrosion, the dissolved oxygen in the system decreases, the influence of metabolites leads to the destruction of biofilms and the continuous attack of metals by chloride ions in the solution.

### EIS measurements and analysis

**Figure 3:**
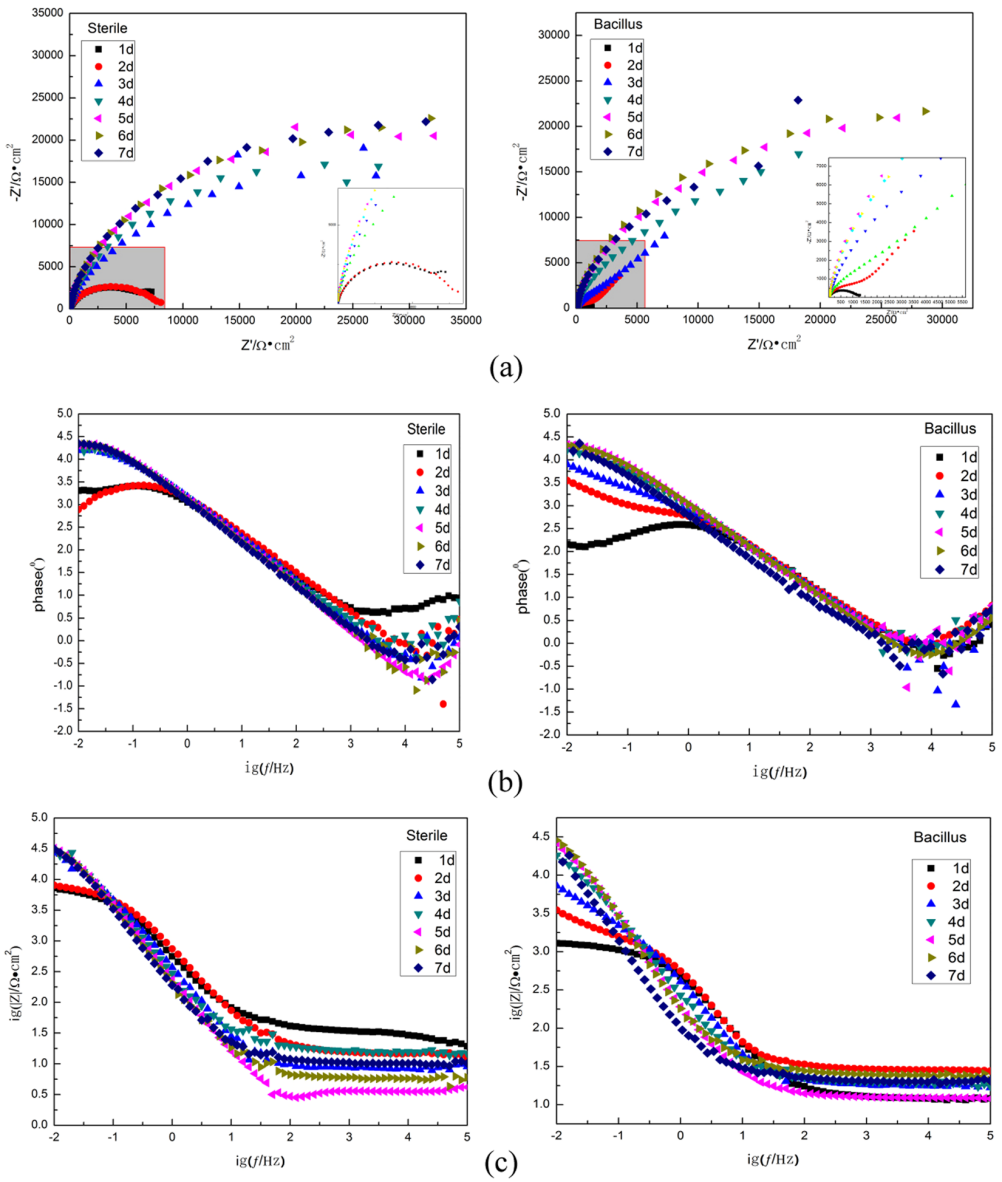
Nyquist and Bode diagrams of 10MnNiCrCu steel immersed in sterile and Bacillus systems for 7d (**a** represents the Nyquist diagram, **b** represents the magnitude diagram, and **c** represents the phase angle diagram).

Figure 3 shows Nyquist and Bode diagrams of 10MnNiCrCu steels immersed in sterile and Bacillus systems for 7 days.

It can be seen from Nyquist diagram that due to frequency dispersion, all Nyquist plots show irregular semicircles, indicating that the corrosion of 10MnNiCrCu steel is controlled by the charge transfer process. The capacitive reactance arc of both systems increase with the immersion time, indicating that the impedance value of the system increases for that the biological/corrosion product film formed on the surface protects the steel temporarily to a certain extent and slows down corrosion. In BS media, as the immersion time increases, the diameter of the semicircle increases first and then decreases, revealing that the corrosion rate decreases and then increases. The decrease of the corrosion rate can be attributed to the formation of the biofilm, while the increase shows the destruction of the film.

As is seen from the Bode diagram, there is two time constant in the sterile system, and the peak values occur at lower frequencies. In Bacillus system, there are two time constants in 2–3d, indicating that it is related to the two-layer structure formed during the corrosion process. The impedance values at low frequencies increased gradually from 1d to 7d, and there were obvious peaks at the lowest frequency in 2-3d. It can be concluded that the structure of bio/corrosion product film formed on the surface of electrode in earlier stage was looser. The capacitance of the product film(Q_b_) decreased during 5-7d, which showed that with the proliferation of BS and the influence of organic acids in metabolites on the product film, the bio/corrosion product film was destroyed and the contact between metal substrate and external corrosive media was further increased, which aggravated the corrosion.

**Figure 4:**
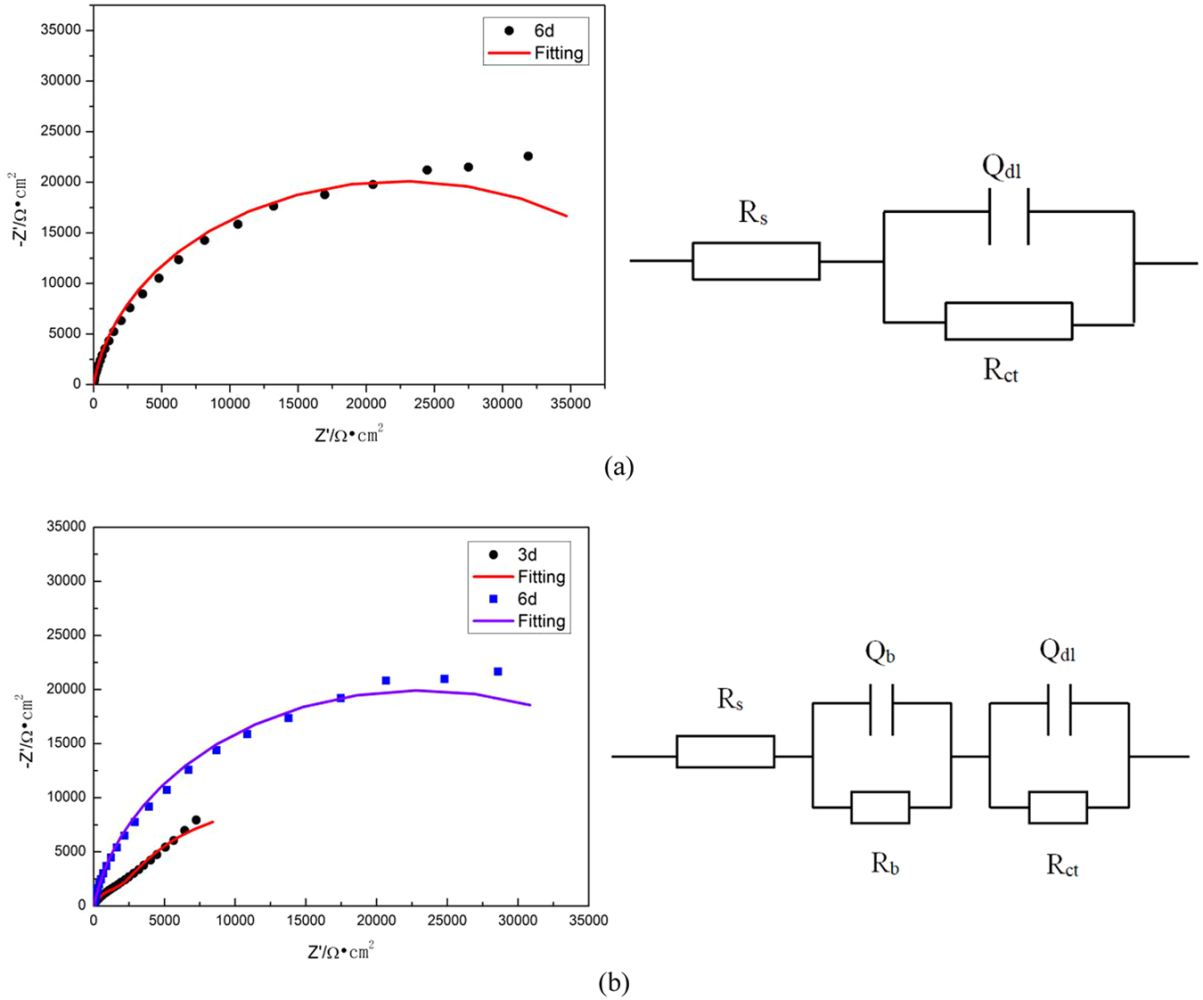
The equivalent circuit models used to fit the EIS experimental data for 10MnNiCrCu steel immersed in (**a**) sterile media and (**b**) BS system.

Considering adsorption of biofilm and corrosion product on the substrate surface and their indistinguishable role, in this study, an equivalent circuit including a quadratic constant as shown in Fig. 4 is used. The sterile system is represented by circuit diagram (a) and the BS system is represented by circuit diagram (b). R_s_ represents the solution resistance, R_b_ represents the protective film resistance on the electrode surface, R_ct_ represents the charge transfer resistance, Q_b_ represents the protective film capacitance, Q_dl_ represents the double layer capacitance, Q represents the constant phase component CPE(when n = 1, CPE = C, that is, the capacitance at this time is the ideal capacitance). The results of fitting parameters are listed in Table 1.

**Table 1:** Parameter fitting values of each element in the equivalent circuit in Fig. 4.

	T (d)	R_s_ (Ω·cm^2^)	Q_dl_ × 10^−4^ (μF·cm^−2^)	n1	R_ct_ (Ω·cm^−2^)	Q_b_ × 10^−4^ (μF·cm^−2^)	n2	R_b_ (Ω·cm^−2^)
Sterile	1	3.07	1.78	0.81	7431	—	—	—
	2	14.33	1.29	0.84	7401			—
	3	8.40	1.34	0.90	32080	—	—	—
	4	15.75	1.41	0.90	37910			—
	5	3.58	1.32	0.94	42030	—	—	—
	6	5.76	1.47	0.93	45270			—
	7	10.14	1.57	0.92	46170	—	—	—
Bacillus	1	11.97	17.69	0.76	709.3	2.45	0.86	577
	2	28.43	14.6	0.72	1102	2.04	0.908	18310
	3	17.74	2.89	0.88	1816	2.89	0.88	22920
	4	19.41	3.27	0.88	39160	4.39	0.90	5875
	5	0.89	3.64	1	42470	1.82	0.90	12.23
	6	5.74	3.51	0.98	46110	1.78	0.91	18.89
	7	0.01	3.12	0.91	20.6	1.78	1	34320

**Figure 5:**
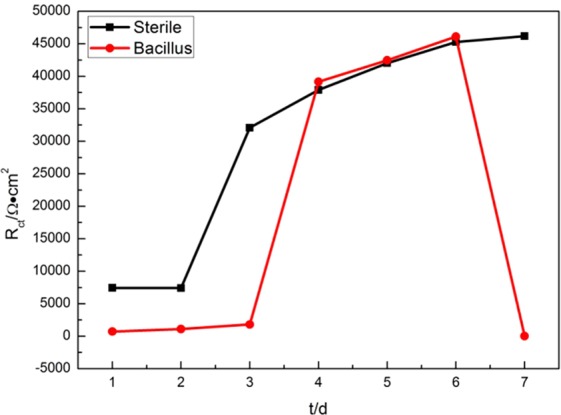
Variation of R_ct_ value over time for 10MnNiCrCu steel immersed in sterile and BS system.

Charge transfer resistance (R_ct_) is an index for evaluating the corrosion rate of metals. Cetin D pointed out that the increase of charge transfer resistance(R_ct_) would increase the porosity of the protective film and Warburg elements are generated, resulting in an increase in the potential of the capacitive element parallel to it^19^. Figure 5 shows the R_ct_ values of the two systems changing with time.

It can be seen from Fig. 5 that the charge transfer resistance R_ct_ in BS system is obviously smaller than that in sterile system, which increased first and then decreased. It not only showed that the corrosion rate of metal surface decreased first and then increased, but also revealed that tendency of the porosity and the surface heterogeneity. The increase of R_ct_ in both systems indicated that a protective film gradually developed during this period. In the Bacillus system, there was a sharp decline within 6–7d, which indicated that the biofilm was destroyed and the corrosion rate of metal was obviously accelerated. In the sterile system, the R_ct_ continuously increased and then achieved a relatively stable state, It showed that the corrosion process of metals in the sterile system was weak.

### Polarization curves

**Figure 6:**
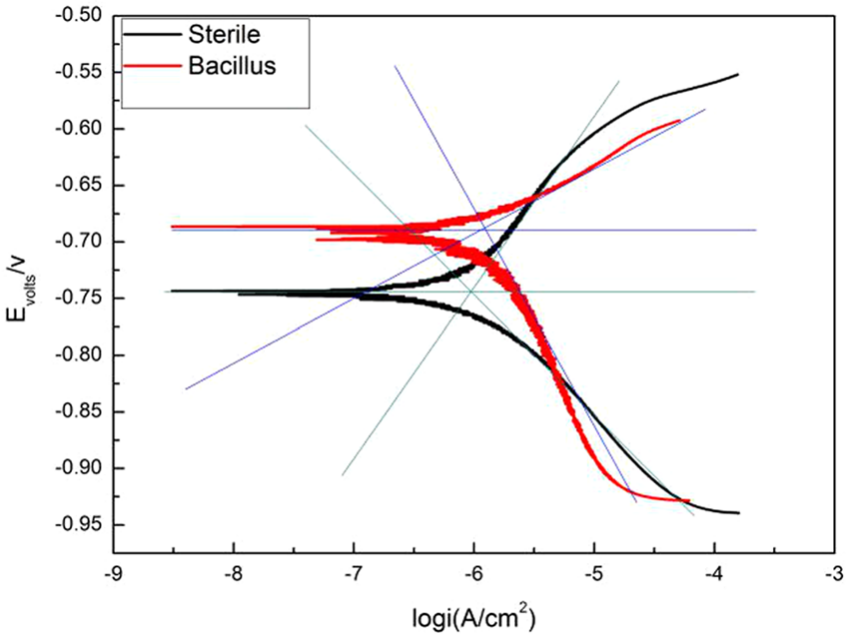
Tafel polarization curve of 10MnNiCrCu steel electrode in sterile and BS system after immersed for 7d.

Figure 6 shows the potentiodynamic polarization curve of 10MnNiCrCu steel immersed in sterile and BS system for 7 days. Tafel curve is fitted by C-view software, and the corresponding electrochemical parameters are obtained, as shown in Table 2. From the figure and the data in the table, it can be seen that a passivation zone occurs in both systems, indicating that a corrosion product layer formed in both systems during the corrosion process. In the presence of microorganisms, a mixed layer of biofilm and corrosion product film may develop in the BS system. In BS system, the slope of cathodic polarization curve is obviously larger than that of anodic polarization curve. It shows that the anode is in the active dissolution state without passivation, and the corrosion reaction is controlled by the cathode^20,21^. With the decrease of dissolved oxygen concentration in the solution, the cathodic reaction is inhibited to some extent. While in the sterile system, the anode slope (158.35 mV·dec^−1^) is slightly larger than the cathode slope (109.57 mV·dec^−1^), indicating that the corrosion reaction is mixed-controlled. According to the fitting data, the corrosion potential of 10MnNiCrCu steel in sterile environment is −1.2272 V, while in the presence of bacteria, the corrosion potential is −1.174 V, and the corrosion current density is 1.0861 μA/cm^2^ and 1.21 μA/cm^2^ respectively. It shows that there are corrosive conditions in both systems. While in the Bacillus system, the passivation area is narrower, indicating that acidic products produced by Bacillus metabolism damage the surface protective film. This is consistent with the results of the discussion and analysis in the impedance section.

**Table 2:** Fitting values of Tafel polarization curves of 10MnNiCrCu steel electrodes in sterile and BS systems.

Condition	E_corr_ (V)	I_corr_ (μA/cm^2^)	B_a_ (mV·dec^−1^)	B_c_ (mV·dec^−1^)
Sterile	−1.2272	1.0861	158.4	109.6
Bacillus	−1.1740	1.2100	64.3	202.5

### Surface morphology analysis with SEM

**Figure 7:**
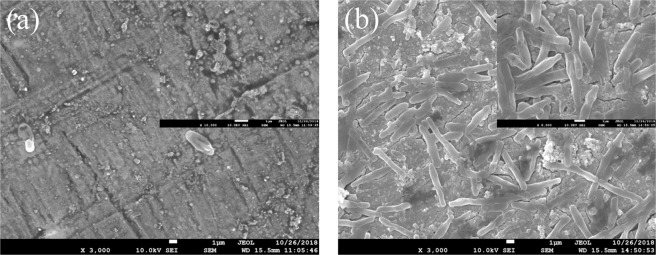
Surface morphology by SEM of 10MnNiCrCu steel immersed in (**a**)sterile medium and (**b**)BS medium for 7 days.

Figure 7 shows the surface morphology of 10MnNiCrCu steel observed by SEM after 7 days immersion in sterile and BS-containing medium, respectively.

It is seen that both of the surfaces of 10MnNiCrCu steel had been subjected to corrosion of different degrees and with different characteristics after immersion, and the corrosion morphology in the two media is quite different. In sterile solution, the metal substrate was relatively complete, and the structure and properties of the internal metal was less affected. In BS solution, a large number of bacteria covered the metal surface and formed a bio/corrosion product film with a loose structure and a great deal of metabolites. Due to the influence of metabolites, cracks appeared on the metal surface and further induced the corrosion extending to the metal interior. Pitting corrosion occurred in the localized area of the metal. Since no mechanism has been established to study the metal corrosion by bacteria by now, it is more consistent to say that metal corrosion is related to the biofilm formed by microorganisms, and the metabolic activity of bacteria is very important for the formation of biofilm. The corrosion area of 10MnNiCrCu steel in BS solution is relatively large, and the pitting corrosion caused by the partial destruction of the corrosion product film is mainly due to the acid and other organic products.

## Discussion

MIC is the corrosion and destruction of materials (including metals and alloys) caused by microorganisms and their EPS involved in the corrosion system. The potential mechanism of MIC can be attributed to the control of protein molecules on the movement of reactants to the metal surface.

In essence, the activity of corrosive microorganisms changes the electrode reaction in the corrosion process. Heyer A reported that the cell walls of bacteria generally showed electronegativity, which would help them adhering to metal surfaces^22^.

In Bacillus system, gas exchange and solution diffusion were blocked in the occluded area, resulting in oxygen deficiency inside and oxygen enrichment outside the area. Thus, the occluded cell generated under biofilm and oxygen concentration corrosion occurred^23^.

1 $${\rm{Fe}}\to {{\rm{Fe}}}^{2+}+2{\rm{e}}$$

2 $${{\rm{H}}}_{2}{\rm{O}}+1/2{{\rm{O}}}_{2}+2{\rm{e}}\to 2{{\rm{OH}}}^{-}$$

The structure of metal substrate was affected by the reaction. Obvious cracks emerged on the metal surface and corrosion was accelerated, for that bacterial metabolites, such as organic acids, destroyed the product film^24,25^.

With the corrosion process, a bio/corrosion product film developed on the metal surface and slowed down the corrosion rate temporarily, as proved by the variation of *E*_*ocp*_. However, the internal redox reaction of metals led to accumulation of a large number of internal ferrous ions.

The chloride ions in the solution were attracted and migrated inside the metal to maintain the charge balance and further promoted the formation of occluded cell. In addition, the biofilm had channels and voids^26^. A large number of Cl^−^ exist whether in sterile or in BS solutions and chloride ions generally accelerate corrosion. In this study, due to the destruction of the film, chloride ions attacked the surface, penetrated the interior of the metal and resulted in the chemical reactions and internal corrosion.

3 $${{\rm{Fe}}}^{2+}+2{{\rm{H}}}_{2}{\rm{O}}+2{{\rm{Cl}}}^{-}\to {\rm{Fe}}{({\rm{OH}})}_{2}+2{\rm{HCl}}$$

4 $${\rm{Fe}}{({\rm{OH}})}_{2}+3{{\rm{Cl}}}^{-}\to {{\rm{FeCl}}}_{3}+2{{\rm{OH}}}^{-}$$

5 $${{\rm{FeCl}}}_{3}+3{{\rm{H}}}_{2}{\rm{O}}\to {\rm{Fe}}{({\rm{OH}})}_{3}+3{\rm{HCl}}$$

The instability of Fe(OH)_3_ in solution could not prevent the metal from corrosion. The continuous entry enabled the rapid increase of internal chloride ions, which had a special effect on anticorrosive metals depending on their passivation film by destroy the film partially and make it incomplete. A large number of metal cations and chloride ions accumulated to form soluble metal chlorides and then hydrolyzed into insoluble metal hydroxides and free acids. The acidic environment with high concentration of chloride ions further promoted the dissolution of internal metals.

**Figure 8:**
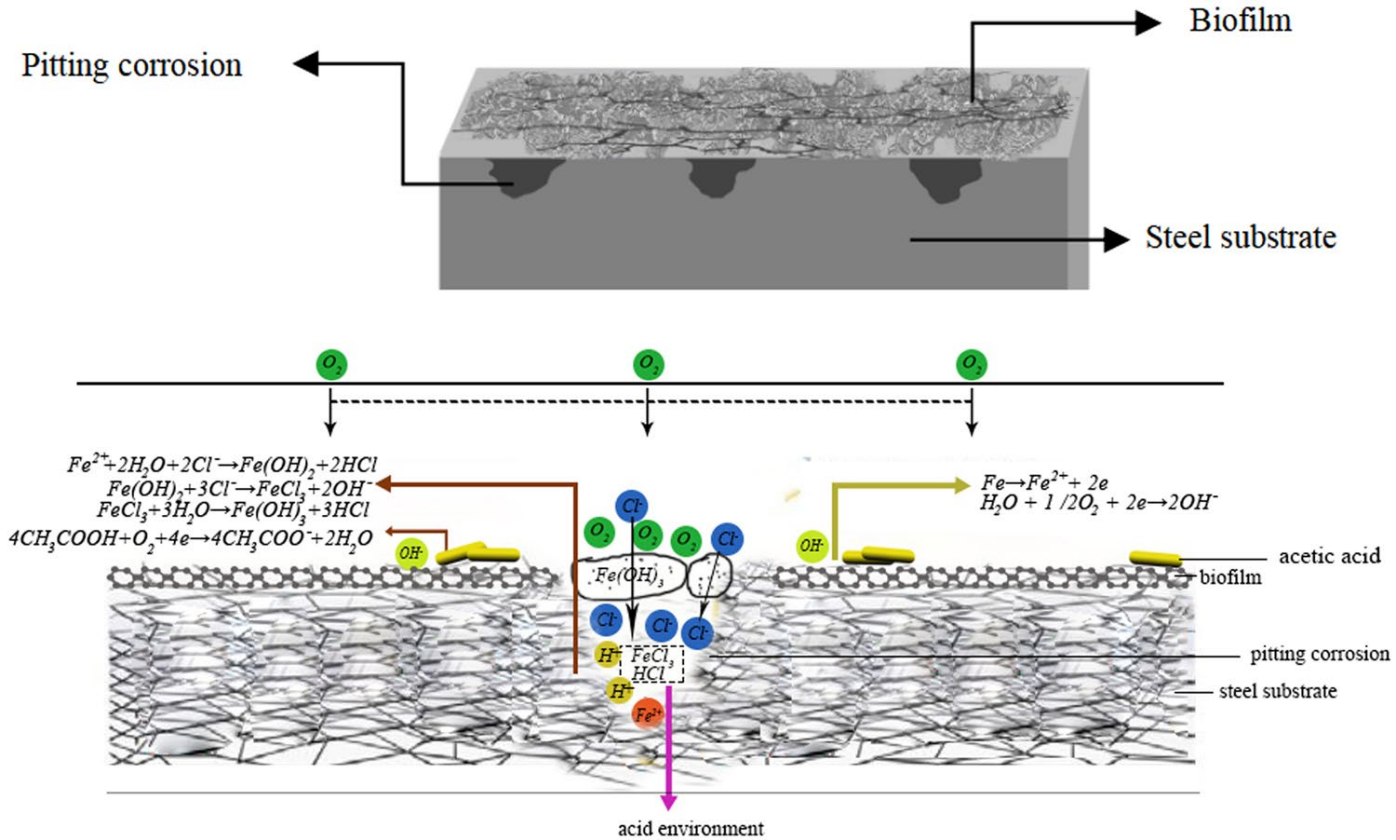
Schematic diagram of the effect of BS on the corrosion process of metals.

In addition, organic acids, the metabolite of bacteria, contained anionic functional groups capable of binding with metals, thus acting as corrosion inducers. The most common low carbon chain fatty acid is acetic acid, which is highly corrosive to steel when concentrated in microbial sediments. Pedersen reported that Pseudomonas promoted passive decomposition by excreting organic acids, resulting in an increase in the corrosion rate of metals^27^. The presence of organic acids accelerated the rate of charge transfer between anode and cathode to a certain extent, thus accelerating the corrosion process^28^. The schematic diagram as shown in Fig. 8 described the effect of BS on the corrosion process of metals.

## Conclusions

The corrosion behavior and mechanism of 10MnNiCrCu steel in media containing *Bacillus subtilis* were studied by growth curve testing, electrochemical analysis and surface morphology observation. The main conclusions of this study are as follows:

In the presence of *Bacillus subtilis*, a bio/corrosion product film with a loose structure and a great deal of metabolites developed on the metal surface due to bacterial adhesion and growth. Due to the propagation of bacteria and the covering of product film, gas exchange and solution diffusion were blocked in the occluded area, then the occluded cell generated under biofilm and oxygen concentration corrosion cells occurred; acidic products in bacterial metabolites such as organic acids contained anionic functional groups capable of binding with metals, which acted as corrosion inducers and destroyed product film, resulting in a large number of obvious cracks on the metal surface; the biofilm provided access for electrolytes and induced the continuous attack of chloride ions on the surface and interior of the metal, leading to pitting corrosion.
